# Gene amplification derived a cancer‐testis long noncoding RNA PCAT6 regulates cell proliferation and migration in hepatocellular carcinoma

**DOI:** 10.1002/cam4.2141

**Published:** 2019-04-09

**Authors:** Shuaizhou Chen, Yao Chen, Qufei Qian, Xuewei Wang, Yuting Chang, Sihan Ju, Yide Xu, Chang Zhang, Na Qin, Hui Ding, Yayun Gu, Jing Han, Cheng Wang, Erbao Zhang, Zhibin Hu

**Affiliations:** ^1^ Department of Epidemiology and Biostatistics Center for Global Health, School of Public Health Nanjing Medical University Nanjing China; ^2^ State Key Laboratory of Reproductive Medicine Nanjing Medical University Nanjing China; ^3^ Jiangsu Key Lab of Cancer Biomarkers, Prevention and Treatment, Jiangsu Collaborative Innovation Center for Cancer Personalized Medicine Nanjing Medical University Nanjing China; ^4^ Department of Bioinformatics, School of Basic Medical Sciences Nanjing Medical University Nanjing China

**Keywords:** cancer‐testis, gene amplification, LIHC, *PCAT6*

## Abstract

Our previous work demonstrated cancer‐testis (CT) genes as a new source of candidate driver of cancer. Recently, mounting evidence indicates that long noncoding RNAs (lncRNAs) with CT expression pattern could play a pivotal role in cancer biology. Here, we characterized a conserved CT long noncoding RNA (CT‐lncRNA), *PCAT6*, which is expressed exclusively in the testis and is reactivated in liver hepatocellular carcinoma (LIHC) tissues due to the highly frequent amplification. The expression in LIHC was correlated with clinical prognosis in TCGA data. Knockdown of *PCAT6* could inhibit cell proliferation and migration in hepatocellular carcinoma (LIHC) cells. Gene set enrichment analysis (GSEA) based on coexpression network revealed that *PCAT6* was involved in similar cilium‐related pathways in the testis and LIHC tissues. However, *PCAT6* was mainly positively correlated with gametogenesis‐related pathways in the testis but was coexpressed with mitotic cell cycle genes in LIHC. Together, our data demonstrated that CT‐lncRNA *PCAT6* represents the similarity and difference between tumorigenesis and gametogenesis. The CT expression pattern and important role in LIHC oncogenesis make *PCAT6* an ideal target for LIHC diagnosis and therapy.

## 1 INTRODUCTION

Liver hepatocellular carcinoma (LIHC) is one of the most common cancers worldwide, accounting for one‐fifth of the incidence of malignant tumors in China.^1^, ^2^ The prognosis of LIHC patients remains very poor, therefore, more research is needed to discover and develop effective biomarkers and targets for LIHC diagnosis and treatment.^3^

Recent genomic studies have revealed that more than 75% of the human genome is transcribed, generating thousands of noncoding RNAs (ncRNAs) with limited or no protein‐coding capacity.^4^, ^5^ Long noncoding RNA (lncRNAs) are operationally defined as RNA transcripts that are >200 nt with limited protein‐coding potential.^8^, ^9^ LncRNA expression profiles are important markers for diverse human cancers.^10^, ^11^ Recently, several lncRNAs have been found to play extensive roles in regulating tumorigenesis including LIHC.^14^ For example, higher expression of lncRNA *CASC9* could affect AKT signaling and DNA damage sensing in LIHC by binding to HNRNPL.^15^

Cancer‐testis (CT) genes are a class of genes with highly restricted expression patterns in normal tissues and are reactivated in a wide range of human tumor types.^16^ The existence of these genes reflects the similarities between the process of gametogenesis and tumorigenesis and provides valuable insight into understanding of tumorigenesis.^16^ Our previous work systematically defined CT genes in 19 cancer types and investigated their potential driving role in carcinogenesis.^17^ Interestingly, recent studies showed that lncRNAs that had CT expression patterns could also contribute to tumorigenesis.^17^, ^18^ For example, lncRNA THOR with a classic CT expression pattern played an important role in the development of lung cancer.^18^ Our previous study has systematically identified long noncoding RNAs with cancer‐testis expression patterns in 14 cancer types.^20^

In this study, we systematically evaluated the expression pattern of previously defined high‐confidence testis‐specific lncRNAs in TCGA LIHC samples 17 and estimated their correlations with copy number level to identify cancer‐related CT‐lncRNAs. We identified a conserved CT‐lncRNA, prostate cancer‐associated transcript 6 (*PCAT6*), as a candidate driver in the development of LIHC. We found that *PCAT6* was significantly up‐regulated in LIHC tissues and predicted a poorer survival in TCGA data. Knockdown of *PCAT6* in LIHC cell lines could inhibit cell growth and migration. Coexpression analysis revealed that *PCAT6* was mainly positively correlated with gametogenesis‐related pathways in testis but was coexpressed with mitotic cell cycle genes in LIHC.

## 2 MATERIALS AND METHODS

### 2.1 The selection of candidate cancer‐testis long noncoding RNAs

The expression and copy number data of LIHC samples were retrieved from The Cancer Genome Atlas project to select the candidate cancer‐testis long noncoding RNAs (CT‐lncRNAs). We obtained the expression data of 371 LIHC samples from the UCSC Xena website (https://xenabrowser.net/datapages/) and downloaded the copy number data of 370 LIHC samples from https://gdac.broadinstitute.org/. Three hundred and sixty‐four LIHC samples with both expression data and copy number data available were included to select the candidate cancer‐testis long noncoding RNAs (CT‐lncRNAs). According to GENCODE v19 annotation data, lncRNAs are reclassified into six biotypes,^21^ including three prime overlapping ncRNA (lncRNAs located within the 3' UTR of protein‐coding genes), antisense (lncRNAs overlapping any protein‐coding genes on the opposite strand), lincRNA (long intergenic noncoding RNA with a length greater than 200 bp), sense intronic (lncRNAs located within the intron of any protein‐coding gene), sense overlapping (lncRNAs within any protein‐coding gene within its intron on the coding strand) and processed transcript (transcripts without an open reading frame). Thus, the high‐confidence testis‐specific long noncoding genes defined in our previous study 17: (1) that exhibited expression (FPKM ≥ 1, Fragments Per Kilobase of exon model per Million mapped fragments) in at least 50% of the LIHC samples; and (2) exhibited amplification in at least 10% of the samples were selected. LncRNAs without copy number data were accessed by the nearest protein‐coding genes (within 10 kb).

### 2.2 RNA extraction, reverse transcription, and the quantitative real‐time PCR

Total RNA was extracted from cells using TRIzol reagent (Invitrogen, Carlsbad, CA) according to the manufacturer's protocol. For qRT‐PCR, 1µg of RNA from each sample was reverse transcribed to complementary DNA (cDNA) by using a Reverse Transcription Kit (Takara, Dalian, China). Real‐time PCR analyses were performed with SYBR PCR Master Mix reagent kit (Takara, Dalian China). Results were normalized against the endogenous expression of GAPDH. The primer sequences are summarized in Table S3.

### 2.3 Cell culture

LIHC cell lines HepG2 and SMMC‐7721 were purchased from the Institute of Biochemistry and Cell Biology of the Chinese Academy of Sciences (Shanghai, China). Cells were cultured in DMEM (GIBCO‐BRL) medium. All media were supplemented with 10% fetal bovine serum (FBS), 100 U/mL penicillin and 100 μg/mL streptomycin. The cells were grown in humidified air at 37°C with 5% CO2.

### 2.4 Transfection of cell lines

The siRNAs were transfected into LIHC cells using Lipofectamine2000 (Invitrogen) according to the manufacturer's instructions. Scrambled negative control siRNA (si‐NC) were purchased from Invitrogen (Invitrogen, CA). The sequences for siRNAs are listed in Table S3.

### 2.5 Cell proliferation assay

Cell viability was measured using MTT kit (Sigma) according to the manufacturer's instructions. For colony formation assay, a certain number of transfected cells were placed in each well of 6‐well plates and maintained in proper media containing 10% FBS for two weeks, during which the medium was replaced every 4 days. Colonies were then fixed with methanol for 20 minutes and stained with crystal violet (Beyotime) for 15 minutes. Colony formation was determined by counting the number of stained colonies. The experiment was repeated three times. EdU assay was determined by using 5‐ethynyl‐2′‐deoxyuridine (EdU) with Cell‐Light^TM^ EdU Apollo 567 in Vitro Kit (Ribobio, Guangzhou, China) according to the manufacturer’s instructions. The EdU incorporation rate was expressed as the ratio of EdU‐positive cells (red cells) to total DAPI‐positive cells (blue cells), which were counted using Image‐Pro Plus (IPP) 6.0 software (Media Cybernetics).

### 2.6 Cell migration assays

After transfection, 4 × 10^4^ cells in serum‐free media were placed in the upper chamber of an insert (8‐μm pore size; Millipore, Billerica, MA). Medium containing 10% FBS was added to the lower chamber. After incubation for 24 hours, the cells remaining on the upper membrane were removed with cotton wool. Cells that had migrated through the membrane were stained with methanol and 0.1% crystal violet, imaged, and counted using an IX71 inverted microscope (Olympus, Tokyo, Japan). Experiments were independently repeated three times.

### 2.7 Co‐expression analysis

The coexpression analysis of *PCAT6* and 20 345 protein‐coding genes defined in GENCODE dataset (https://www.gencodegenes.org/, Version 19) was performed using Spearman rank sum test to avoid skewness. The expression data of testis were downloaded from the Genotype‐Tissue Expression (GTEx) portal (https://gtexportal.org/home/datasets, GTEx Version 7). We used the false discovery rate (FDR) for multiple statistical tests.

### 2.8 Gene set enrichment analysis

The gene set enrichment analysis (GSEA) was applied in LIHC and testis samples based on the GO Biological Process Ontology gene sets with the R Bioconductor package clusterProfiler. Only gene sets with more than 20 genes were included in this analysis. All protein‐coding genes were ranked based on the correlation coefficient derived from the above coexpression analysis, and gene sets with *P* < 1.60 × 10^5^ (0.05/3116) were included in the following analysis.

### 2.9 Survival analysis

The clinical data of 342 LIHC samples were obtained from the data portal of Genomic Data Commons (GDC, https://gdc-portal.nci.nih.gov/legacy-archive/). A multivariate Cox proportional hazards regression model was used to estimate the hazard ratios (HR) and their 95% confidence intervals (CIs) with adjustment for age, gender, and clinical stage. K‐M method was used to create the survival plots. All the analyses were implemented by the R package “survival”.

### 2.10 Evaluation of the association of *PCAT6* and cancer stemness

We performed a correlation analysis between *PCAT6* and *CD34* with Spearman rank sum test to avoid skewness in 371 LIHC samples. The expression data were downloaded from the *UCSC Xena website (* https://xenabrowser.net/datapages/ *).* We also computed Spearman correlations of *PCAT6* against mRNA expression‐based stemness index (mRNAsi) and DNA methylation‐based stemness index (mDNAsi) separately in 368 LIHC samples. The two stemness indices for each LIHC sample were downloaded from the NIH Genomic Data Commons (GDC, https://gdc.cancer.gov/about-data/publications/PanCanStemness-2018).

### 2.11 Statistical analysis

Spearman rank sum test was used to evaluate the association of expression level with the copy number of *PCAT6* in 364 LIHC samples. Paired *t* test was used for the differential expression analysis of log2 transformed expression data of *PCAT6* in 50 tumor/adjacent paired samples. Two‐sides *P *> 0.05 were considered as statistically significant. General statistical analyses were performed with R software (R version 3.5.1).

## 3 RESULT

### 3.1 Integration of gene expression and copy number data identified a conserved CT‐lncRNA *PCAT6* as the candidate driver for LIHC

To identify candidate CT‐lncRNAs contributing to the development of liver hepatocellular carcinoma (LIHC), we integrated gene expression and copy number data of 364 LIHC samples from the TCGA project. Firstly, we identified six long noncoding genes with high abundance (FPKM ≥ 1 in more than 50% samples) in LIHC tissues from high‐confidence testis‐specific noncoding genes defined in our previous study (Figure S1).^17^ Among the six noncoding genes, 10.44% (38/364) of the patients showed amplification in *PCAT6* (Table S1).

**Figure 1:**
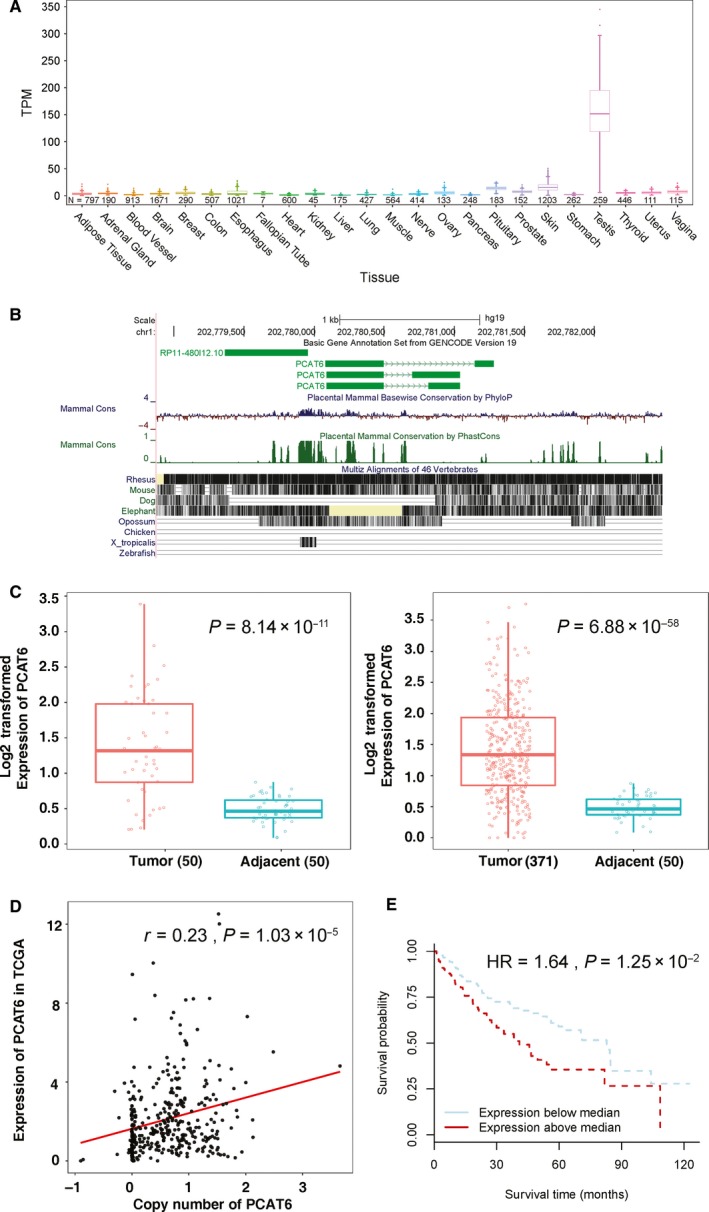
The selection of *PCAT6* as a candidate driver CT‐lncRNA in liver hepatocellular carcinoma. (A) Expression of *PCAT6* in 24 types of normal tissues from the GTEx portal. (B) Genomic annotation of *PCAT6* with UCSC Genome Browser (hg19/GRCh37). (C) Expression of *PCAT6* in TCGA LIHC samples was significantly higher than that in adjacent samples (paired and unpaired tissues). (D) Expression of *PCAT6* in TCGA LIHC samples was positively correlated with its copy number level. (E) Higher expression of PCAT6 was significantly associated with a poor overall survival in TCGA LIHC samples

*PCAT6* was a CT‐lncRNA with typical restricted expression pattern in the testis (Figure 1A) and showed high conservation in multiple vertebrates (Figure 1B). In order to evaluate the potential function of *PCAT6* in LIHC, we further performed differential expressed analysis in TCGA data. The result revealed that the expression of *PCAT6* was significantly higher in LIHC samples than adjacent samples (Paired Wilcoxon rank sum test: *P* = 8.14 × 10^−11^; Unpaired Wilcoxon rank sum test: *P* = 6.88 × 10^−58^; Figure 1C), and the expression of *PCAT6* in LIHC samples was significantly correlated with the copy number level (Cor = 0.23, *P* = 1.03 × 10^−6^; Figure 1D). Additionally, Cox regression analysis showed that increased expression of *PCAT6* was significantly associated with a poorer overall survival (HR = 1.64, *P* = 1.25 × 10^−2^; Figure 1E).

### 3.2 *PCAT6* regulates LIHC cell proliferation and migration

**Figure 2:**
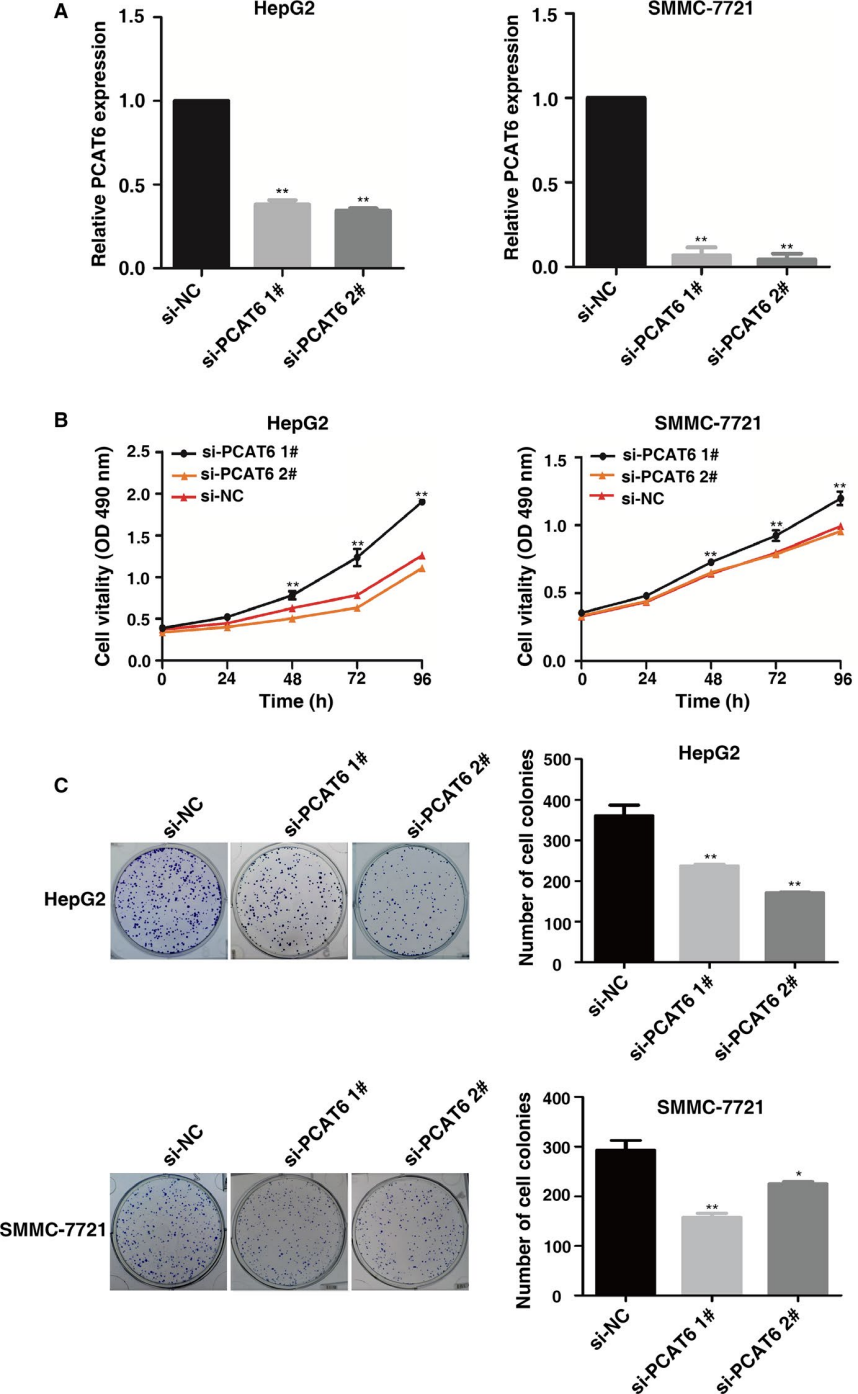
PCAT6 regulates LIHC cell proliferation. (A) qRT‐PCR was performed to detect PCAT6 expression after siRNA‐mediated knockdown and plasmid‐mediated overexpression in HepG2 and SMMC‐7721 cells. (B) MTT assays were performed to determine cell proliferation of HepG2 and SMMC‐7721 cells after transfection. (C) Colony formation assays of HepG2 and SMMC‐7721 cells after transfection. *P < 0.05, **P < 0.01

To explore the role of *PCAT6* in LIHC, we used siRNA targeting *PCAT6*. As shown in Figure 2A, the siRNA‐mediated knockdown was used for exogenous repression expression of *PCAT6* in HepG2 and SMMC‐7721 cells. It was shown that after transfection with the specific siRNA, HepG2 and SMMC‐7721 cells exhibited significantly decreased expression level of *PCAT6*.

**Figure 3:**
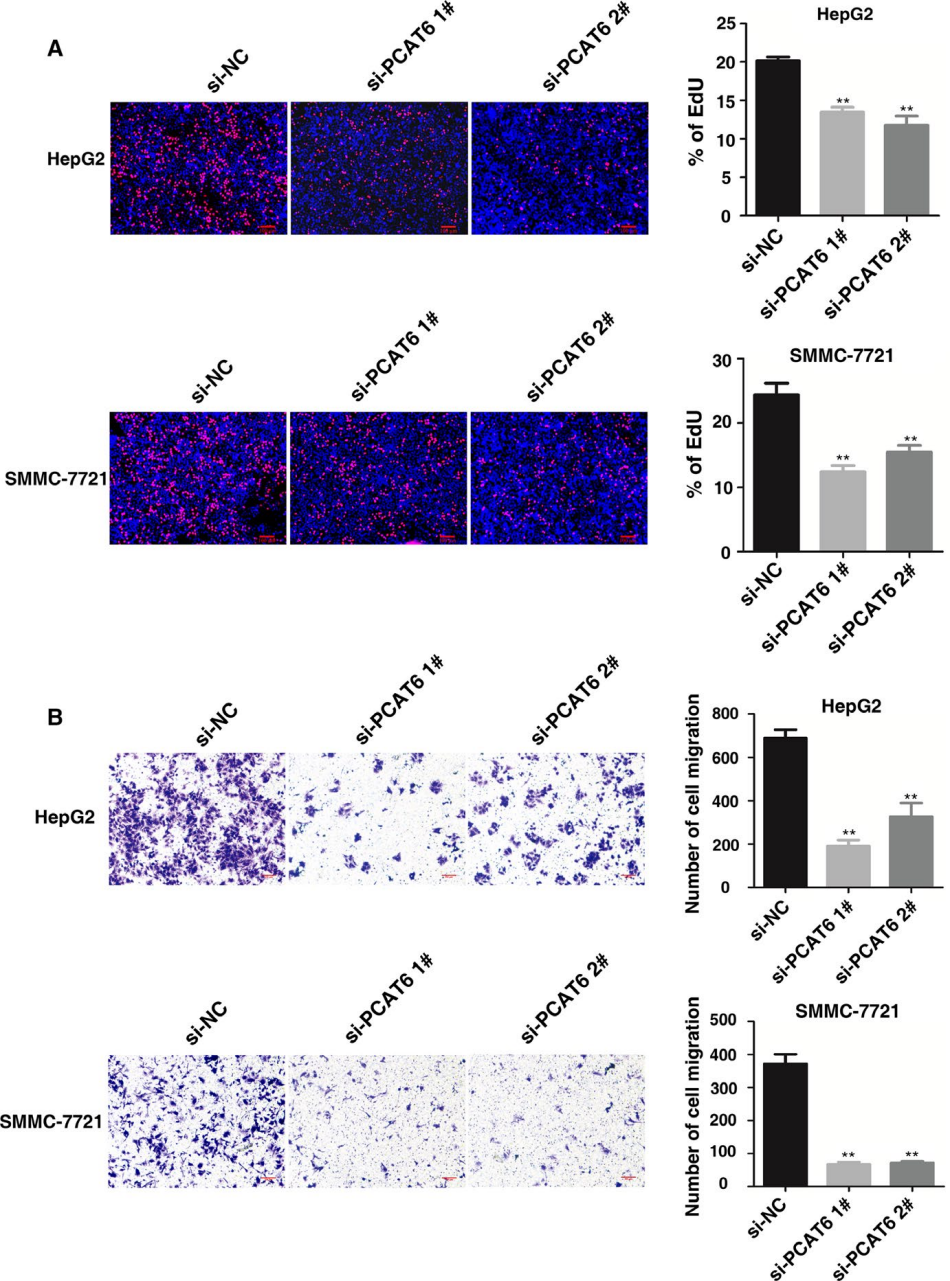
PCAT6 regulates LIHC cell proliferation and migration. (A) EdU assays of HepG2 and SMMC‐7721 cells after transfection. (B) Transwell assays were used to investigate the migratory abilities of HepG2 and SMMC‐7721 cells after transfection. *P < 0.05, **P < 0.01

Then MTT assays showed that knockdown of *PCAT6* expression significantly inhibited cell proliferation compared with the control cells (Figure 2B). Similarly, the result of colony formation assay revealed that clonogenic survival was significantly decreased following knockdown of *PCAT6* (Figure 2C). EdU assays showed that *PCAT6* had a significant impact on cell proliferation in HepG2 and SMMC‐7721 cells (Figure 3A). Next, transwell assays revealed that knockdown of *PCAT6* significantly repressed cell migration compared with the control both in HepG2 and SMMC‐7721 cells (Figure 3B).

### 3.3 Functional prediction of *PCAT6* based on coexpressed protein‐coding genes

**Figure 4:**
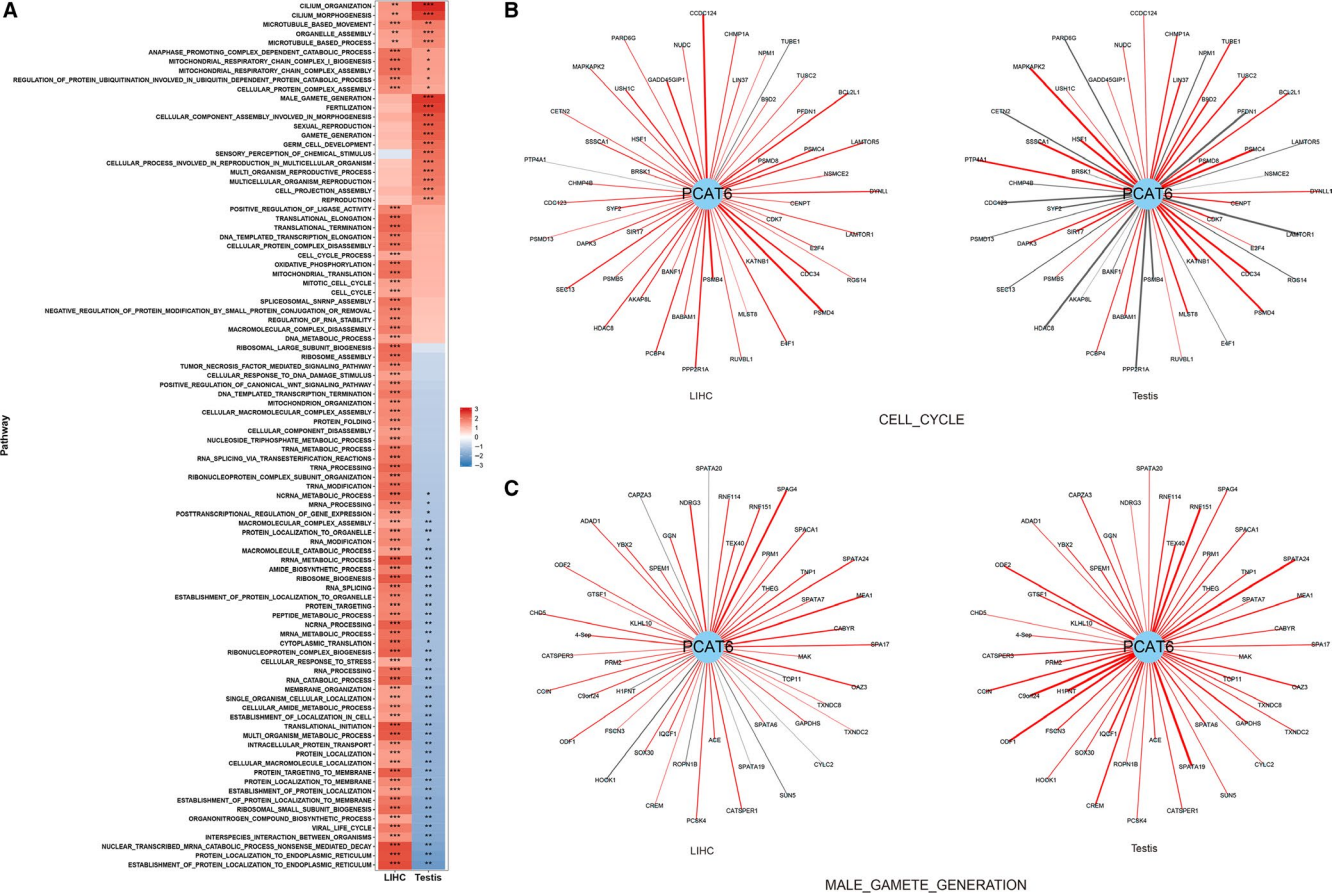
Coexpression analysis and gene set enrichment analysis (GSEA) of *PCAT6.* (A) Heatmap of normalized enrichment score (NES) derived from GSEA of *PCAT6* in 371 LIHC samples and 259 testis samples. ***P < 1.60 × 10^−5^; **P < 0.01; *P < 0.05. (B) The coexpression pattern of *PCAT6* and genes in the cell cycle pathway in LIHC and testis samples, respectively. The lead 50 coexpressed genes in LIHC samples are presented. Red line denotes positive correlation and gray line denotes negative correlation. The width of the line denotes the absolute value of the correlation coefficient. (C) The coexpression patterns of *PCAT6* and genes in the male gamete generation pathway in LIHC and testis samples, respectively. The lead 50 coexpressed genes in testis samples are presented. Red line denotes positive correlation and gray line denotes negative correlation. The width of the line denotes the absolute value of the correlation coefficient

To explore the different functions of *PCAT6* in tumorigenesis and spermatogenesis, we applied Gene Set Enrichment Analysis (GSEA) on the correlation coefficients from coexpression analysis with 20 345 protein‐coding genes in 371 LIHC samples and 259 testis samples, respectively. A total of 94 gene sets were identified as significant (*P* < 0.05/3116) in either testis or LIHC tissues (Figure 4A, Table S2). Interestingly, genes positively coexpressed with *PCAT6* in the LIHC and testis samples were consistently enriched in the same pathways, including cilium‐related gene sets (Figure 4A), suggesting that these regulatory networks of *PCAT6* were involved in gametogenesis and tumorigenesis of LIHC in a similar way. However, the enrichment status of 87.23% (82/94) gene sets varied across testis and LIHC tissues: 10 gene sets were specifically enriched in the testis, 31 gene sets were specifically enriched in LIHC, and 41 positively enriched gene sets in LIHC samples were negatively enriched in the testis samples (Figure 4A). For example, genes coexpressed with *PCAT6* were positively enriched in cell cycle related gene sets in LIHC but were more prone to be enriched in male gamete generation related gene sets in testis (Figure 4A). Additionally, we observed that genes involved in the above two pathways showed extremely different coexpression correlations with *PCAT6* in the LIHC and testis samples, respectively (Figure 4B). Among the top 50 cell cycle genes coexpressed with *PCAT6* in LIHC samples, 48 were positively coexpressed with *PCAT6* and nearly 21 genes showed negative correlations with *PCAT6* in the testis samples, such as E4F1 (Figure 4B). Similarly, among the lead 50 coexpressed genes in the male gamete generation pathway in testis, all of them were positively correlated with *PCAT6* and nine genes showed negative correlations in the LIHC tissues (Figure 4C). To further evaluate the oncogenic role of PCAT6, we performed a correlation analysis to explore the relationship between *PCAT6* and stemness. However, we found that the expression of *PCAT6* showed a significantly negative association with *CD34*, a marker of stemness (Figure S2A) (r = −0.12, *P* = 1.97 × 10^−2^).^22^ Additionally, no relationship was observed between *PCAT6* and mRNAsi (mRNA expression‐based stemness index)/mDNAsi (DNA methylation‐based stemness index) (r = −0.07, *P* = 1.76 × 10^−1^; r = 0.03, *P* = 5.71 × 10^−1^; Figure S2B).

## 4 DISCUSSION

The processes of germ cell formation and tumorigenesis share important similarities, including immortalization, induction of meiosis and metastasis.^16^ CT genes are characterized by highly restricted expression patterns in normal tissues and reactivated expression in a wide range of human tumor types.^16^ CT genes have been identified to play essential roles in cancer cell survival and may serve as potential therapeutic targets including LIHC.^23^, ^24^ For example, CT gene DUSP21 could regulate tumorigenesis of LIHC both in vitro and in vivo.^25^ In a recent study, it was found that lncRNA THOR with a classic CT expression pattern, defined as one of the new class of lncRNAs could also contribute to the development of multiple cancer types.^18^ Our previous study also showed that CT‐lncRNA LIN28B‐AS1 could regulate cell proliferation and migration by binding to IGF2BP1 in lung cancer.^19^

In our present study, we revealed that PCAT6 was a new CT‐lncRNA of LIHC. Its activation in LIHC tissues was associated with a poor prognosis and the knockdown of PCAT6 could also significantly inhibit cell proliferation and migration both in HepG2 and SMMC‐7721 cells. Several recent studies also presented the important role of PCAT6 in other cancers. High PCAT6 was positively correlated with tumor size, TNM stage, and lymph node metastasis in lung cancer tissues.^28^ Knockdown of PCAT6 could repress lung cancer cell proliferation and migration.^29^ Moreover, PCAT6 was up‐regulated in colon cancer and PCAT6 promoted cell growth and inhibited cell apoptosis by binding to EZH2.^30^

In addition, we found that gene amplification of PCAT6 could induce the overexpression of PCAT6 in LIHC. The copy number amplification of oncogenes is widely considered as the driver of cancer.^31^ However, most studies focused on the protein‐coding genes in the amplification regions. Our data suggested that the gene amplification of lncRNA PCAT6 could partially explain the activation of PCAT6 in tumorigenesis of LIHC, emphasizing the importance of lncRNA in the copy number altered regions. A previous study has also shown that a gain of copy number could induce the expression of lncRNA PVT1 in tumorigenesis.^32^ The results also suggested that the copy number amplification may also serve as an activation mechanism of CT‐lncRNAs.

Interestingly, our coexpression enrichment analysis further demonstrated that CT‐lncRNA PCAT6 represents the similarity and difference between tumorigenesis and gametogenesis. We found that PCAT6 could be involved in the same pathways in the LIHC and testis, such as cilium organization/morphogenesis. In addition to the similarity, we also found the difference tumorigenesis and gametogenesis. The activation of PCAT6 in LIHC co‐occurred with a number of genes in mitotic cell cycle and RNA processing, which were also the hallmark pathway of cancer. And PCAT6 co‐expressed genes in testis were mainly having a crucial role in gametogenesis and reproduction. These results indicated the importance of PCAT6 in both tumorigenesis and spermatogenesis, which warrants further investigation in the future.

In summary, our results showed that PCAT6 was identified as functional CT‐lncRNA in the tumorigenesis of LIHC. Its CT expression pattern and important role in LIHC oncogenesis make it an ideal target for LIHC diagnosis and therapy.

## CONFLICT OF INTEREST

The authors declare that they have no competing interests.
