# Supplementary figures and images for: Gene amplification derived a cancer‐testis long noncoding RNA PCAT6 regulates cell proliferation and migration in hepatocellular carcinoma
Source: Cancer Med. 2019 Apr 9;8(6):3017–25. doi: 10.1002/cam4.2141 (PMC6558594; doi:10.1002/cam4.2141)

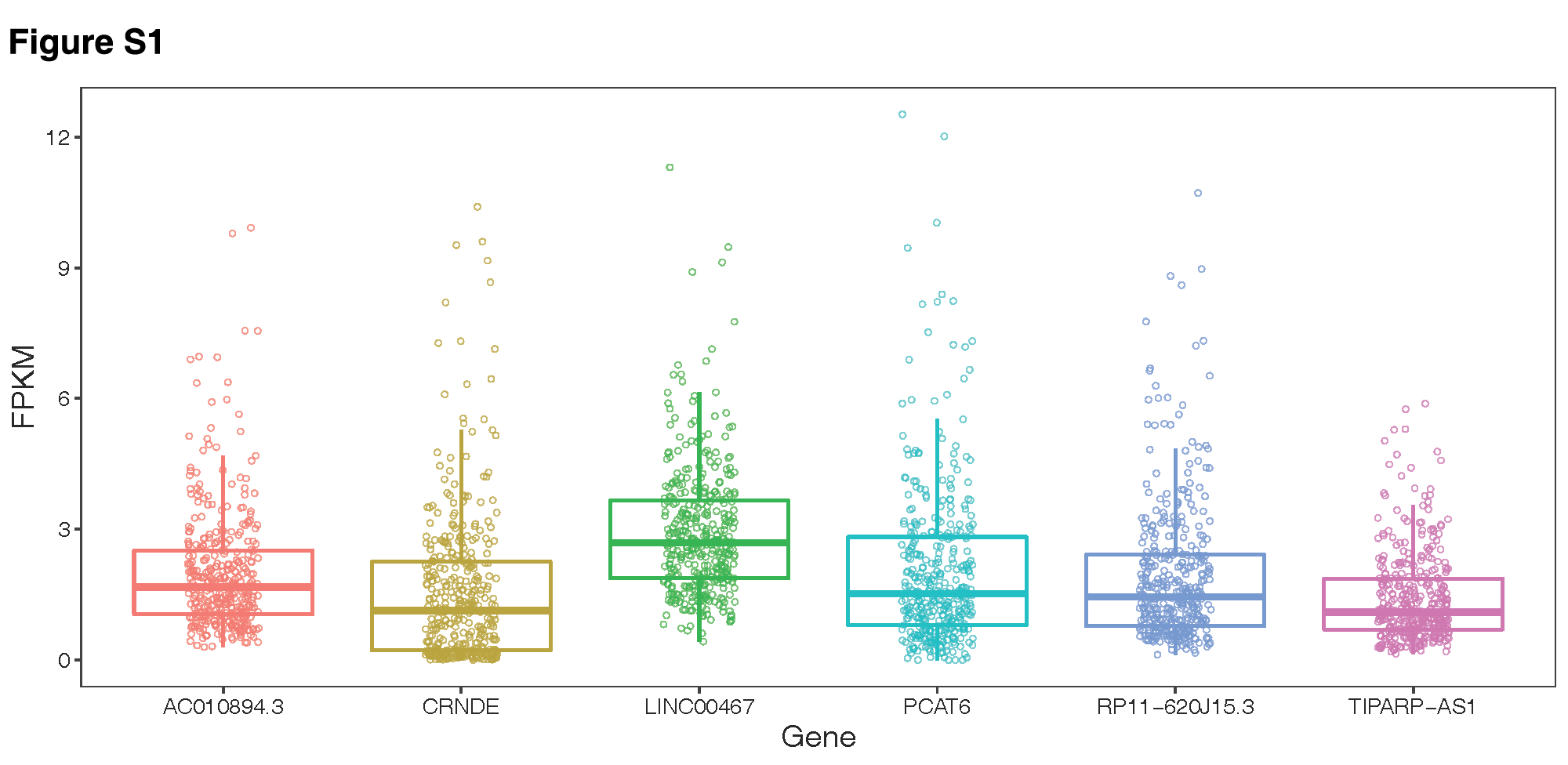

Supplement: Supplementary file 1 [file CAM4-8-3017-s001.tif]

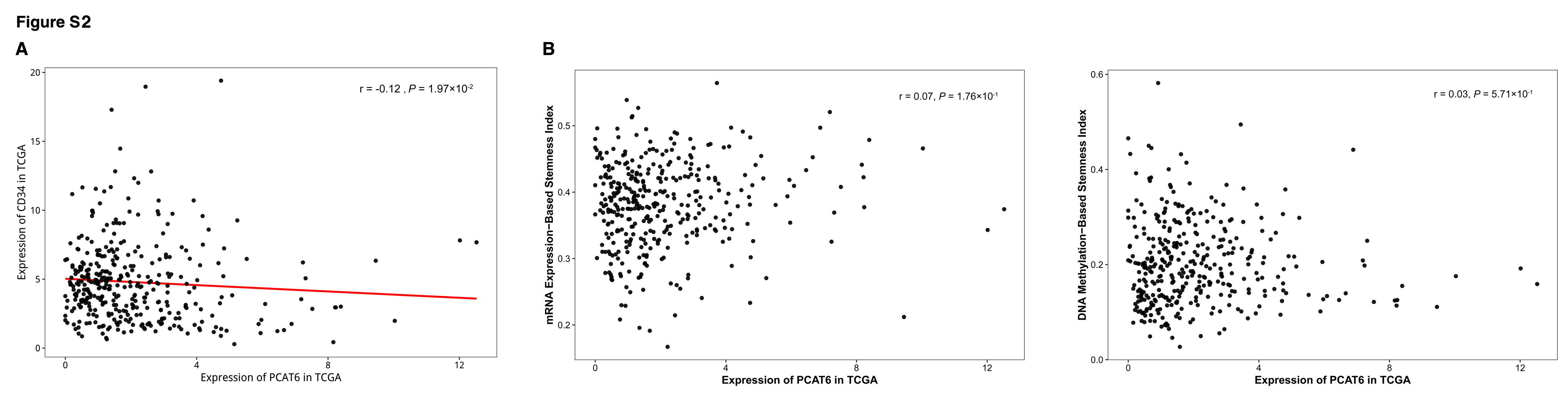

Supplement: Supplementary file 2 [file CAM4-8-3017-s002.tif]
